# Pairwise association of upper extremity musculoskeletal conditions: large population investigation from PERSIAN cohort study

**DOI:** 10.1186/s13018-023-04108-6

**Published:** 2023-08-26

**Authors:** Mahla Daliri, Farideh Khosravi, Mohammad-T. Shakeri, Mohammad H Ebrahimzadeh, Ali Moradi

**Affiliations:** 1 Orthopedics Research Center, Ghaem Hospital, Mashhad University of Medical Sciences, Mashhad, 91388-13944 Iran; 2 Student Research Committee, School of Health, Mashhad University of Medical Sciences, Mashhad, Iran; 3 Department of Biostatistics, Mashhad University of Medical Sciences, Mashhad, Iran

**Keywords:** Carpal tunnel syndrome, Lateral epicondylitis, Musculoskeletal, Pairwise association, Upper extremity

## Abstract

**Background:**

People with one area of upper extremity musculoskeletal conditions (UEMSCs) may have other. We aim to determine how frequent is the ipsilateral coexistence of common UEMSCs apparent on interview and examination.

**Methods:**

This is a large population cross-sectional study conducted as part of the PERSIAN cohort study int Mashhad University of Medical Sciences (MUMS). The study aimed to evaluate individuals for symptoms and signs of the following conditions: carpal tunnel syndrome (CTS), lateral epicondylitis (LE), trapeziometacarpal osteoarthritis (TMC OA), DeQuervain’s tendinopathy, trigger digit (TD), ganglion cyst, and rotator cuff tendinopathy (RCT). The primary outcomes of the study are (1) to determine the side-specific relative risk of each UEMSC coexisting with the second condition, and (2) to identify predictive factors of each UEMSC using side-specific multivariate logistic regression analysis.

**Results:**

We conducted a study involving 4737 individuals from the staff of MUMS and found significant pairwise associations among UEMSCs on a side-specific basis. Women had more chance of having DeQuervain’s disease (*β* = 6.3), CTS (*β* = 3.5), ganglion cyst (*β* = 2.5), TMC OA (*β* = 2.3), and RCT (*β* = 2.0). Each condition (dependent variable) was associated with others (predictors) as follows: CTS: RCT (*β* = 5.9), TMC OA (*β* = 4.7), TD (*β* = 2.9), and LE (*β* = 2.5). TMC OA: LE (*β* = 6.4), TD (*β* = 5.4), RCT (*β* = 4.3), and CTS (*β* = 4.1). LE: RCT (*β* = 8.1), TMC OA (*β* = 6.4), and CTS (*β* = 2.5). DeQuervain’s disease: TD (*β* = 13.6), RCT (*β* = 4.5), and LE (*β* = 3.8). TD: CTS (*β* = 8.8), ganglion cyst (*β* = 7.6), DeQuervain’s disease (*β* = 5.7), and TMC OA (*β* = 4.3). RCT: LE (*β* = 5.8), TMC OA (*β* = 5.5), CTS (*β* = 5.2), and DeQuervain’s disease (*β* = 4.3). Ganglion cyst: TD (*β* = 4.8).

**Conclusion:**

Our study reports significant increased frequency of the UEMSCs among patients who already have one of the diseases, in a large sample size study.

*Level of Evidence* Level II (Differential Diagnosis/Symptom Prevalence Study).

## Introduction

Prevention of upper extremity musculoskeletal conditions (UEMSCs) is of significance through understanding their common risk factors. Tendinitis, synovitis, tenosynovitis, osteoarthritis, DeQuervain’s disease, epicondylitis, and carpal tunnel syndrome are common UEMSCs. Epidemiological research has discovered strong correlations between these diseases and physical risk factors causing strain [1–3].

Carpal tunnel syndrome (CTS), a widely recognized condition with a high prevalence, has prompted investigations into potential association with other upper extremity conditions [4]. CTS has been found to exhibit associations with lateral epicondylitis (LE) [5, 6], shoulder and cervical pain [7, 8], and has notably been observed to co-occur with LE in surgically treated patients [9]. LE, commonly known as tennis elbow, demonstrates correlations with DeQuervain’s disease, CTS [9, 10], and rotator cuff tendinopathy (RCT) [11], potentially indicating shared underlying mechanisms. Biomechanical factors, such as prolonged non-neutral wrist posture, may account for the relationship between CTS and conditions like LE [12]. Additionally, there is another study reporting notable relationships between CTS and trapeziometacarpal osteoarthritis (TMC OA) [13]. The presence of CTS alongside TMC OA in surgical cases underscores the need for proactive identification and simultaneous treatment to minimize postoperative complications. Furthermore, shared risk factors prompt the investigation of trigger digit’s intriguing association with CTS, revealing genetic insights that shed light on potential localized mechanisms [14]. In light of these complex association networks, the study hypothesizes an evaluation of the concurrent occurrence of these upper extremity musculoskeletal conditions.

Shared local [15], psychosocial [16, 17], biomechanical (occupational) [12, 18], metabolic [19], and genetic [14, 20] factors, as well as a previous history of upper extremity injury, are hypothesized underlying contributors to the concomitant UEMSCs. By estimating the likelihood that two UEMSCs would develop concurrently, we may focus on high-risk individuals and use physical examinations for early detection and secondary prevention (screening). We herein study the 4737 personnel of the PERSIAN Cohort Study in Mashhad University of Medical Sciences (MUMS) who underwent clinical assessment (history and physical examination) for UEMSCs, to investigate the likelihood of concurrent two upper limb conditions in a large population of healthcare staff. Doing this cross-sectional study, we inquire about the relative risk of common UEMSCs for the second disease existence in side-specific basis.

## Methods

### Setting and population

**Fig. 1:**
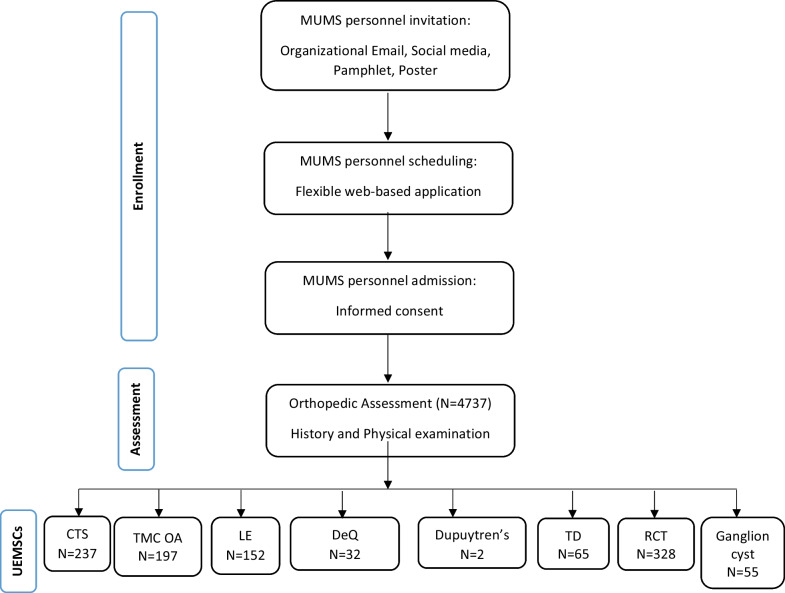
Population enrollment flowchart

This study uses the data gathered in (Removed due to Blinding). The study protocol has received approval from the institutional review board of the (Removed due to Blinding), and adheres to the criteria outlined in the Helsinki declaration. Prior to the study, all participants provide their informed consent, and they are free to decide at any time whether to continue participating or not. Patients enrollment process and study design are reported here [21] in details (Fig. 1).

### Study design

During a cross-sectional study, one trained orthopedic surgery resident PGY 2/4 (who received training from Dr. A.M., the senior hand surgeon, before the commencement of the study) took history and did physical examination of enrolled individuals to detect the upper extremity conditions, including CTS, LE, trapeziometacarpal joint osteoarthritis (TMC OA), DeQuervain’s disease, trigger digit (TD), ganglion cyst, and rotator cuff tendinopathy (RCT). We made a brief self-reported questionnaire and a physician clinical assessment for the orthopedic evaluation of the PERSIAN Cohort Study in Mashhad University of Medical Sciences (MUMS). Diagnosis were made clinically based on history and physical examination, as described in Table 1. Each criterion related to a history question or a diagnostic physical examination test has 1 score. For each condition, diagnosis was made if the person fulfilled all the determined criteria (Table 1). For each condition, people were dichotomized into two groups of “Yes” for those who diagnosed having the disease (positive for all criteria), “No” for those who diagnosed not having the disease (negative for all criteria). Suspicious cases that we could not diagnose the disease or not (positive for some criteria) were excluded from the study.

**Table 1:** Diagnosis criteria

Condition	Questions/test	History	Physical exam	Diagnosis score
Carpal tunnel syndrome	1. Hand paresthesia and tingling, primarily in the thumb, index digit, middle digit, and half of the ring digit (the thumb side)	1		3
	2. Pain and paresthesia during night sleep that wakes the patient up	1		
	3. Positive Compression test^1^		1	
Lateral epicondylitis	1. Pain on the outside of the elbow especially while doing activities like turning a door handle or opening a jar	1		2
	2. Presence of direct tenderness over lateral epicondyle		1	
DeQuervain’s tendinitis	1. Wrist pain specially when doing something that involves grasping or pinching	1		3
	2. Positive Finkelstein test^2^		1	
	3. Presence of tenderness over the first extensor compartment		1	
Trigger digit	1. Presence of triggering in digits		1	2
	2. Point tenderness over volar side of metacarpophalangeal joints		1	
Ganglion cyst	1. Presence of a round, cystic, prominent mass in the digits, hand, and wrist		1	1
Trapeziometacarpal arthritis	1. Pain at the base of the thumb especially when writing, turning a door key, or using spoon	1		3
	2. Point tenderness at the base of the thumb in the first carpometacarpal joint		1	
	3. Positive Grind test^3^		1	
Rotator cuff tendinopathy	1. Pain in anterolateral shoulder	1		4
	2. Pain during night sleep that wakes the patient up	1		
	3. Pain with forward elevation and internal rotation	1		
	4. Positive Hawkins test^4^		1	

^1^Compression test: The examiner placed direct pressure for 30 s over the carpal tunnel (median nerve) between the thenar and hypothenar eminence while the patient’s forearm was in the supination position. Any numbness or paresthesia in the median nerve’s distribution was indicated as a positive test result. This test is 87% sensitive and 95% specific for CTS

^2^Finkelstein test: The Finkelstein test is the standard provocative test for determining the presence of DeQuervain’s disease. The patient was instructed to make a fist by actively flexing their thumb to the maximum extent. In order to stretch the muscles in the first extensor compartment, the patient then deviates his or her wrist to the ulnar side. If the patient reports pain in the wrist’s first extensor compartment, the test is considered positive

^3^Grind test: The thumb grind test is used to evaluate TMC joint arthritis and the health of the basal joint. The examiner conducted the grind test by holding the patient’s thumb’s metacarpal bone and rotating it in a circle while applying light axial force. A typical complaint from a patient with thumb joint arthritis is a sudden, acute pain at the CMC joint, which may also be accompanied by crepitus; this indicates a positive test

^4^Hawkins test: The examiner places the patient’s arm shoulder in 90 degrees of shoulder flexion with the elbow flexed to 90 degrees and then internally rotates the arm. The test is considered to be positive if the patient experiences pain with internal rotation. This test is 62–92% sensitive, and 25–100% specific for rotator cuff tendinopathy (impingement syndrome)

### Descriptive data

The mean age of the study population was 43.5 years, and men-to-women ratio was close to 1. Most of the individuals were married (86.7%), with bachelor degree (45.1%). Nurses and midwives comprised the highest proportion of the population, accounting for 19% (Table 2).

**Table 2:** Population demographic data (*N* = 4737)

Variable	Result
Sex	
Men	2205 (46.5)
Women	2532 (53.5)
Age (year)	43.5 ± 8.78 (23–83)
Education Level	
Elementary	93 (2.0)
Middle school	112 (2.4)
Diploma	582 (12.3)
Associate degree	430 (9.1)
Bachelor	2138 (45.1)
Master	744 (15.7)
PhD or PhD equivalent	631 (13.3)
Illiterate	7 (0.1)
Education Years	16.30 ± 3.82
Marital status	
Single	400 (8.4)
Married	4105 (86.7)
Widow	68 (1.4)
Divorced	155 (3.3)
Others	9 (0.2)
Occupation	
Nurse and midwife	888 (19)
Officer	711 (15)
Medical assistants	396 (8.4)
Educational experts	382 (8.1)
Technicians	266 (5.6)
Nurse assistant	131 (2.8)
Has fracture history in prior 5 years	165 (3.5)

### Statistical analysis and outcomes

We used SPSS software (version 26) to perform statistical analysis. Descriptive statistics were reported as mean, standard deviation (SD), and prevalence (%). The relative risk (RR), its *p* value, and 95% confidence interval were calculated. Statistical significance was two-tailed at *p* value < 0.05. The primary outcomes of the study are (1) to determine the side-specific relative risk of each UEMSC coexisting with the second condition, and (2) to identify predictive factors of each UEMSC using side-specific multivariate logistic regression analysis. To answer these, we considered limbs as the samples (each individual as two samples).

## Results

### Diseases prevalence

According to Fig. 1, RCT had the most prevalence (6.9 percent) among studied musculoskeletal conditions.

#### Carpal tunnel syndrome (CTS)

The RR for all upper extremity conditions was significant among patients with CTS, except for ganglion cyst and Dupuytren’s contracture. Women have 3.54 times more chance to have CTS. Individuals with LE, TMC OA, RCT, and TD are 2.5, 4.7, 6, and 2.9 times more likely to have concomitant CTS on their ipsilateral limb, respectively (Table 3).

**Table 3:** Carpal tunnel syndrome (CTS)

Ipsilateral association of CTS with other upper limb orthopedic conditions (*N* = 377)			
Condition	Prevalence (%)	95% CI	RR (*P* value)
Rotator cuff tendinopathy	25.2	7.19–10.89	8.85 (< 0.001)
TMC OA	18.0	8.18–14.04	10.72 (< 0.001)
Lateral epicondylitis	13.0	6.99–13.23	9.62 (< 0.001)
Trigger digit	6.1	8.15–22.5	13.55 (< 0.001)
DeQuervain’s disease	4.2	14.07–56.54	28.21 (< 0.001)
Ganglion cyst	1.3	0.80–4.96	1.99 (0.13)

Multivariate analysis for ipsilateral association of CTS with other upper limb orthopedic conditions (*N* = 377)			
Condition	Exp (Beta)	95% CI	*P* value
Gender (female/male)	3.54	2.62–4.77	< 0.001
Lateral epicondylitis	2.55	1.53–4.27	0.005
TMC OA	4.77	3.14–7.26	< 0.001
Rotator cuff tendinopathy	5.98	4.36–8.21	< 0.001
Trigger digit	2.94	1.36–6.34	0.006

#### TMC osteoarthritis (TMC OA)

The RR for all upper extremity conditions was significant among patients with TMC OA, except for ganglion cyst and Dupuytren’s contracture. Women have 2.31 times more chance to have TMC OA. Every one-unit increase in the age year led to 1.02-times increase in TMC OA existence. Individuals with LE, CTS, RCT, DeQuervain’s, and TD are 6.4, 4.1, 4.3, 17.2, and 5.4 times more likely to have concomitant TMC OA on their ipsilateral limb, respectively (Table 4).

**Table 4:** Trapeziometacarpal osteoarthritis (TMC OA)

Ipsilateral association of TMC OA with other upper limb orthopedic conditions (*N* = 253)			
Condition	Prevalence (%)	95% CI	RR (*P* value)
Rotator cuff tendinopathy	29.2	7.1–10.7	8.7 (< 0.001)
Carpal tunnel syndrome	26.9	7.2–11.2	9.01 (< 0.001)
Lateral epicondylitis	22.1	10.8–19.1	14.37 (< 0.001)
DeQuervain’s disease	9.9	53.29–204.4	104.38 (< 0.001)
Trigger digit	9.5	9.84–24.7	15.6 (< 0.001)
Ganglion cyst	1.2	0.53–5.3	1.68 (0.37)

Multivariate analysis for ipsilateral association of TMC OA with other upper limb orthopedic conditions (*N* = 253)			
Condition	Exp (Beta)	95% CI	*P* value
Gender (female/male)	2.31	1.44–3.68	< 0.001
Age (year)	1.02	1.00–1.04	0.035
Lateral epicondylitis	6.47	3.35–12.49	< 0.001
Carpal tunnel syndrome	4.1	2.42–6.94	< 0.001
Rotator cuff tendinopathy	4.3	2.56–7.22	< 0.001
DeQuervain’s disease	17.2	5.71–51.81	< 0.001
Trigger digit	5.45	1.89–15.70	0.002

#### Lateral Epicondylitis (LE)

The RR for all upper extremity conditions was significant among patients with LE, except for ganglion cyst and Dupuytren’s contracture. Women have 1.62 times more chance to have LE. Every one-unit increase in the age year led to 1.03-times increase in LE existence. Individuals with TMC OA, RCT, and CTS are 6.4, 8.1, and 2.5 times more likely to have concomitant LE on their ipsilateral limb, respectively (Table 5).

**Table 5:** Lateral epicondylitis (LE)

Ipsilateral association of LE with other upper limb orthopedic conditions (*N* = 198)			
Condition	Prevalence (%)	95% CI	RR (*P* value)
Rotator cuff tendinopathy	35.4	9.1–13.5	11.1 (< 0.001)
TMC OA	28.3	10.25–17.29	13.32 (< 0.001)
Carpal tunnel syndrome	24.7	6.13–10.25	7.93 (< 0.001)
Trigger digit	8.1	7.89–19.88	11.71 (< 0.001)
DeQuervain’s disease	7.1	21.0–76.1	40.04 (< 0.001)
Ganglion cyst	0.5	0.09–5.0	0.7 (0.72)

Multivariate analysis for ipsilateral association of LE with other upper limb orthopedic conditions (*N* = 198)			
Condition	Exp (Beta)	95% CI	*P* value
Gender (female/male)	1.62	1.05–2.52	0.028
Age (year)	1.03	1.02–1.06	< 0.001
TMC OA	6.4	3.83–10.70	< 0.001
Rotator cuff tendinopathy	8.11	5.18–12.71	< 0.001
Carpal tunnel syndrome	2.5	1.48–4.20	0.001

#### DeQuervain’s disease (DeQ)

The RR for all upper extremity conditions was significant among patients with DeQuervain’s disease, except for Dupuytren’s contracture. Women have 6.3 times more chance to have DeQuervain’s disease. Individuals with LE, TD, RCT, and TMC OA are 3.8, 13.6, 4.6, and 22.7 times more likely to have concomitant DeQuervain’s disease on their ipsilateral limb, respectively (Table 6).

**Table 6:** DeQuervain’s disease (DeQ)

Ipsilateral association of DeQ with other upper limb orthopedic conditions (*N* = 37)			
Condition	Prevalence (%)	95% CI	RR (*P* value)
TMC OA	67.6	30.4–52.5	39.9 (< 0.001)
Rotator cuff tendinopathy	48.6	10.6–20.0	14.57 (< 0.001)
Carpal tunnel syndrome	43.2	10.3–21.3	14.87 (< 0.001)
Lateral epicondylitis	37.8	17.8–43.9	28.0 (< 0.001)
Trigger digit	27.0	26.6–87.6	48.28 (< 0.001)
Ganglion cyst	5.4	2.1–32.9	8.35 (0.002)

Multivariate analysis for ipsilateral association of DeQ with other upper limb orthopedic conditions (*N* = 37)			
Condition	Exp (Beta)	95% CI	*P* value
Gender (female/male)	6.3	1.41–28.08	0.016
Lateral epicondylitis	3.8	1.26–11.47	0.018
Trigger digit	13.64	4.18–44.48	< 0.001
Rotator cuff tendinopathy	4.58	1.69–12.44	0.003
TMC OA	22.7	8.67–59.42	< 0.001

#### Trigger digit (TD)

Except for Dupuytren’s contracture, the RR of all conditions was significant among patients with TD. Every one-unit increase in the age year led to 1.05-times increase in TD existence. Individuals with CTS, TMC OA, DeQuervain’s, and ganglion cyst are 8.8, 4.3, 5.7, and 7.6 times more likely to have concomitant TD on their ipsilateral limb, respectively (Table 7).

**Table 7:** Trigger digit (TD)

Ipsilateral association of TD with other upper limb orthopedic conditions (*N* = 80)			
Condition	Prevalence (%)	95% CI	RR (*P* value)
TMC OA	30.0	8.6–17.6	12.3 (< 0.001)
Carpal tunnel syndrome	28.7	6.6–13.1	9.33 (< 0.001)
Rotator cuff tendinopathy	26.3	4.4–9.2	6.42 (< 0.001)
Lateral epicondylitis	20.0	6.5–16.3	10.32 (< 0.001)
DeQuervain’s disease	12.5	27.9–108.9	55.17 (< 0.001)
Ganglion cyst	3.8	1.74–16.8	5.4 (0.004)

Multivariate analysis for ipsilateral association of TD with other upper limb orthopedic conditions (*N* = 80)			
Condition	Exp (Beta)	95% CI	*P* value
Age (year)	1.05	1.02–1.09	< 0.001
Carpal tunnel syndrome	8.8	4.29–18.08	< 0.001
TMC1 OA	4.34	1.62–11.58	0.003
DeQuervain’s disease	5.76	1.64–20.19	0.006
Ganglion cyst	7.63	1.65–35.20	0.009

#### Rotator cuff tendinopathy (RCT)

The RR of all upper extremity conditions was significant among patients with RCT, except for ganglion cyst and Dupuytren’s contracture. Every one-unit increase in the age year led to 1.03-times increase in RCT existence. Women have 2 times more chance to have RCT. (Table 8). Individuals with TMC OA, CTS, LE, and DeQuervain’s are 5.5, 5.1, 5.8, and 4.3 times more likely to have concomitant RCT on their ipsilateral limb, respectively (Table 8).

**Table 8:** Rotator cuff tendinopathy (RCT)

Ipsilateral association of RCT with other upper limb orthopedic conditions (*N* = 427)			
Condition	Prevalence (%)	95% CI	RR (*P* value)
Carpal tunnel syndrome	22.2	7.3–11.3	9.13 (< 0.001)
TMC OA	17.3	8.4–14.4	11.04 (< 0.001)
Lateral epicondylitis	16.4	12.4–22.8	16.83 (< 0.001)
Trigger digit	4.9	5.1–13.9	8.4 (< 0.001)
DeQuervain’s disease	4.2	15.6–64.1	31.7 (< 0.001)
Ganglion cyst	1.2	0.6–4.1	1.65 (0.27)

Multivariate analysis for ipsilateral association of RCT with other upper limb orthopedic conditions (*N* = 427)			
Condition	Exp (Beta)	95% CI	*P* value
Gender (female/male)	2.04	1.55–2.69	< 0.001
Age (year)	1.03	1.01–1.04	< 0.001
TMC1 OA	5.58	2.75–7.62	< 0.001
Carpal tunnel syndrome	5.19	3.61–7.46	< 0.001
Lateral epicondylitis	5.88	3.41–10.12	< 0.001
DeQuervain’s disease	4.38	1.56–12.28	0.005

#### Ganglion cyst

The RR for TD and DeQuervain’s disease was 5.4 and 8.5, respectively (*P* value = 0.003) among patients with ganglion cyst. Women have 2.57 times more chance to have ganglion cyst. Individuals with TD are 4.8 times more likely to have concomitant ganglion cyst on their ipsilateral limb (Table 9).

**Table 9:** Ganglion cyst (GC)

Ipsilateral association of GC with other upper limb orthopedic conditions (*N* = 68)			
Condition	Prevalence (%)	95% CI	RR (*P* value)
Carpal tunnel syndrome	7.4	0.8–4.4	1.92 (0.12)
Rotator cuff tendinopathy	7.4	0.7–3.7	1.6 (0.26)
TMC OA	4.4	0.5–5.0	1.66 (0.37)
Trigger digit	4.4	1.7–16.6	5.4 (0.003)
DeQuervain’s disease	2.9	2.1–34.6	8.5 (0.003)
Lateral epicondylitis	1.5	0.1–4.9	0.7 (0.072)

Multivariate analysis for ipsilateral association of GC with other upper limb orthopedic conditions (*N* = 68)			
Condition	Exp (Beta)	95% CI	*P* value
Gender (female/male)	2.57	1.48–4.46	0.001
Trigger digit	4.86	1.48–15.87	0.009

## Discussion

The primary objective of our study was to assess the concurrent presence of musculoskeletal conditions in the upper extremity. Conducting a large population-based study, we aimed to investigate the pairwise associations among clinically diagnosed upper extremity musculoskeletal conditions and their prevalence patterns. Notably, our findings reveal a substantial association among the clinically diagnosed UEMSCs.

Among the studied UEMSCs, RCT emerges as the most prevalent condition, with a prevalence of approximately 7%, consistent with prior literature [22]. RCT’s prominence is also reflected in its status as the most commonly identified work-related upper extremity (UE) condition in a systematic review [23]. Risk factors for this condition includes age, dominant arm, and trauma history [24]. We observed significant associations between RCT and CTS, as individuals with RCT are nine times more likely to have CTS. This association is also observed in another study reporting patients experiencing right-sided RCT having a notable odds ratio of 2.12 for right-sided CTS [25].

We have found that among people with CTS, the RR for LE and RCT is 9.6 and 8.8, respectively. The intricate relationships within the UEMSCs network extend to the structural and biomechanical dimensions. Wee TC et al. provide insights into shared pathophysiology, as common extensor tendon (CET) sonographic abnormalities in CTS patients without clinical symptoms of LE suggest a potential association [26]. Moreover, the biomechanical implications of prolonged non-neutral wrist posture, as observed in lateral epicondylitis (LE), raise questions about the potential bidirectional influence between CTS and other orthopedic conditions [12]. LE reported in 33% (vs. 13% in our study) of patients with surgically treated CTS [9]. Among risk factors for women with CTS, tennis elbow had the OR of 1.73 (95% CI 1.34–2.22) [27]. Among 512 manual laborers, CTS prevalence reported as 6.6 percent, and this prevalence increased among those with rotator cuff tendinopathy and epicondylitis to 13.3% (vs. 22% in our study) and 20.7% (vs. 25% in our study), respectively [25]. However, it is also possible that CTS is the initiating condition that causes the onset of other problems. TD and CTS frequently coexist, linked by shared systemic risk factors like diabetes, rheumatoid arthritis, and hypothyroidism. A substantial percentage of patients with idiopathic trigger digit also exhibit CTS [19]. Recent genome-wide association studies emphasize genetic connections, specifically implicating the DIRC3 gene and IGF-1 signaling, underlining the localized mechanisms contributing to their co-occurrence [14].

The concept of “Basal joint pain syndrome” is proposed to address the complex interplay between TMC OA and associated skeletal or soft tissue diseases, highlighting the need for concurrent treatment [28]. In fact, 65% of patients required at least one additional surgical procedure, and 75% received therapy for associated musculoskeletal issues before, during, or after arthroplasty surgery [28]. It is worth noting that maintaining prolonged and specific postures, such as in professions like dentistry, is associated with thumb osteoarthritis [29]. Similar to our results, CTS showed to be 39% (vs. 27% in our study) prevalent among patients who surgically treated for TMC OA [13]. The reason could be the conjunction of bone architectural changes in CTS and reduced space owing to flexor tenosynovitis. Given the notable association, special effort should be taken to detect or rule out coexisting carpal tunnel syndrome in patients scheduled for basal joint surgery so that, if present, it can be treated concurrently, reducing the risk of postoperative morbidity and delayed symptoms.

### Limitations

This study possesses several strengths, notably a substantial sample size and comprehensive clinical evaluations. However, it is not without its limitations. Para-clinical tests of imaging or electro-physiologic study were not conducted to offer additional confirmation for the musculoskeletal problems. However, most of the included diseases typically are diagnosed clinically in practice, and further investigations were neither financially nor time-wise possible. We suspect the excessively high association between TMC OA and DeQuervain’s disease is due to overlap and similar diagnosis criteria, as well as our evaluator’s (orthopedic resident) lack of experience distinguishing between these two conditions. Being a cross-sectional study, present and prior research on this topic cannot indicate which of these conditions develop first. Although this study included large population, some diseases were positive in a small number of people like DeQuervain’s disease, limiting the analysis sample size. It is unknown whether the medical university and hospital personnel who registered for the study are representative of the whole general community, and it is probable that those with medical conditions were more willing to engage in this investigation.

The observation of significant co-occurrence of musculoskeletal conditions within the upper extremity among non-care seeking individuals underscores the importance of employing comprehensive care for both diagnosing and treating these musculoskeletal conditions.

## Data Availability

The datasets used and/or analyzed during the current study are available from the corresponding author on reasonable request. Correspondence and requests for materials should be addressed to MoradiAL@mums.ac.ir.
